# The Expanding Role of Electrospray Ionization Mass Spectrometry for Probing Reactive Intermediates in Solution

**DOI:** 10.3390/molecules171011507

**Published:** 2012-09-27

**Authors:** Weitao Zhu, Yu Yuan, Peng Zhou, Le Zeng, Hua Wang, Ling Tang, Bin Guo, Bo Chen

**Affiliations:** 1 Key Laboratory of Chemical Biology and Traditional Chinese Medicine Research (Ministry of Education of China), Hunan Normal University, 36 Lushan Road, Changsha 410081, China; Email: weitao0329@126.com (W.Z.); zhou_peng@189.cn (P.Z.); zengle8899@126.com (L.Z.); hiction@hotmail.com (H.W.); tanya051423@tom.com (L.T.); dr-chenpo@vip.sina.com (B.C.); 2 School of Pharmaceutical Sciences, Central South University, 172 Tongzipo Road, Changsha 410013, China; Email: csuyy@yahoo.com.cn

**Keywords:** intermediates, reactive metabolite, electrospray, ambient ionization, mass spectrometry, chemical trapping, solution

## Abstract

Within the past decade, electrospray ionization mass spectrometry (ESI-MS) has rapidly occupied a prominent position for liquid-phase mechanistic studies due to its intrinsic advantages allowing for efficient “fishing” (rapid, sensitive, specific and simultaneous detection/identification) of multiple intermediates and products directly from a “real-world” solution. In this review we attempt to offer a comprehensive overview of the ESI-MS-based methodologies and strategies developed up to date to study reactive species in reaction solutions. A full description of general issues involved with probing reacting species from complex (bio)chemical reaction systems is briefly covered, including the potential sources of reactive intermediate (metabolite) generation, analytical aspects and challenges, basic rudiments of ESI-MS and the state-of-the-art technology. The main purpose of the present review is to highlight the utility of ESI-MS and its expanding role in probing reactive intermediates from various reactions in solution, with special focus on current progress in ESI-MS-based approaches for improving throughput, testing reality and real-time detection by using newly developed MS instruments and emerging ionization sources (such as ambient ESI techniques). In addition, the limitations of modern ESI-MS in detecting intermediates in organic reactions is also discussed.

## 1. Introduction

In chemistry, reaction intermediates that are generated by for example, pyrolysis, radiolysis, combustion, discharge, bio-activation and degradation are usually high-energy, highly reactive, short-lived and hence elusive molecular species. Studies on these transient species are not exactly a novelty, but they have attracted increasing research interest over the past decades as they can provide crucial insight into the reaction mechanisms [1,2,3], bioactivation-related adverse drug effects [4,5], as well as pollutant transformation process [6]. However, due to the intrinsic properties of these transient species, in particular their reactivities, they can rarely be separated from the reaction solution and thus sometimes are “observable” only by very fast detection techniques to detect their existence in the reaction medium.

The experimental determination of reaction intermediates has traditionally relied on spectroscopic approaches usually including ultraviolet-visible absorption (UV), nuclear magnetic resonance (NMR), Raman, electron spin resonance (ESR), and infrared (IR) spectroscopies. For directly investigating short-lived species related to the chemical reaction, different sophisticated methods have been developed by using cryogenic matrix-spectroscopic techniques [7] or combining them with some special procedures like pulse radiolysis/flash photolysis (fast detection in real time based on high power light pulses), matrix isolation (stabilization by chemical trapping or low temperature) and flow systems (monitoring of steady-state intermediate concentration) [8,9]. Recently more fast spectroscopic methods based on high-speed (pico-through microsecond) spectroscopy have been proposed for directly observing the generation and decomposition of transient species in aqueous solution [10,11].

Despite the great achievements made in the field of fast time-resolved spectroscopic techniques, the classical experiments also suffer from some remarkable limitations for the following reasons: (1) conventional spectroscopic methods typically require the presence or introduction of chromophores (or fluorophores), radiotracers, nuclei (or electrons) with characteristic spin [2]; (2) substrates, intermediates, and products cannot be distinguished and monitored simultaneously due to the problem of overlapping bands in their photo electron spectra [12]; (3) during the reaction progress, sufficient changes of reactants (consumption) → intermediates (formation) → products (accumulation) and their isolation in complex mixtures is typically required to allow for their unambiguous identification and accurate quantification [2]; (4) it seems to be not generally suited to studying reactive intermediates directly in reaction solutions in case of complex reactions, for example, a cascade or radical chain reaction [12,13]. For example, NMR is one of the most powerful and versatile techniques now available for studying molecular structures and reaction mechanisms, but generally it is time-consuming [14]. Clearly, with more demanding challenges of direct (real time), rapid (high-throughput) and simultaneous monitoring of transient intermediate species in reaction processes, exploring alternative analytical techniques to overcome these weaknesses would be of increasing importance for acquiring important new insights in our understanding of reactions and the dynamic mechanisms.

Over the last several decades, mass spectrometry (MS), owing to its outstanding sensitivity, selectivity and speed, has become one of the most promising techniques among analytical tools available to provide structural information (*i.e.*, mass-to-charge ratio, isotopic distribution, fragmentation pattern, ion signal intensity) for intermediates (plus reactants and products) during chemical reactions. Accordingly, the main challenge and the key to success of MS-based method for online monitoring of chemical reactions is designing its interface to allow for real-time recording of the intermediate/product species generated from the reactions [15]. But for most of its history, MS has long served as a leading tool for gas-phase ion chemistry. By classic hard ionization techniques (such as electron impact, chemical ionization, atom bombardment) ions are created under vacuum, in which some highly reactive small (volatile) molecules could be generated and monitored in the gas phase, e.g., femtosecond real-time observation of benzyne intermediates in a molecular beam [16], neutralization-reionization mass spectrometry for neutral intermediates [1,17,18]. Indeed, a few “softer” vacuum ionization mass spectrometers, including secondary ion MS (SIMS) and matrix-assisted laser desorption/ionization mass spectrometry (MALDI-MS), could be used to produce intact molecular ions from condensed-phase samples. Nevertheless, the high vacuum condition often invited skepticism because of the use of eccentric environment with no bridge to the “real world” [19]. 

Fortunately, the breakthrough advances in ionizations at atmospheric pressure instead of *in vacuo*, suited for interfacing the high-vacuum analyzer with the condensed-phase sampling system opened up the access to the direct probing chemical/biological reaction mechanisms by mass spectrometry. In particular, modern MS instruments coupled with atmospheric pressure ionization (API) or an atmospheric pressure version of MALDI (AP-MALDI) [20] offer significant advantages over other well-established spectroscopic techniques employed for the investigation of reaction intermediate species. Both API and MALDI enable the generation of intact molecular ions at atmospheric pressure from a condensed phase (MALDI for solid and API for liquid) to the gas-phase within the vacuum system. While MALDI-MS is proven well suited to mapping out entire molecular weight distribution of larger molecules in processed samples [2], API-MS that can create gas-phase ions from liquid-reacting species/products provides distinct benefits of direct detection of reaction intermediates and real-time monitoring of dynamic process in reaction solutions [21]. The most common API techniques for liquid-based ionization strategy are electrospray ionization (ESI) and atmospheric pressure chemical ionization (APCI). Although ESI-MS is not very well suited for neutral intermediates and non-polar molecules, which can be transferred from the solution (evaporating droplets) to the gas phase and then ionized in APCI via a cascade of ion-molecule reactions (electron abstraction, protonation or/and deprotonation) initiated by corona discharge [3,22,23], it has been rapidly becoming the method of choice for solution mechanistic studies within the past decade [3,21,24,25,26,27] due to its own intrinsic advantages for efficient characterization and direct investigation of reaction intermediates.

In this review we attempt to offer a comprehensive overview of the ESI-MS-based methodologies developed up to date to study the reactive intermediates in reaction solution. A full description of the general issues involved in probing intermediate species in complex reaction systems, such as the main sources of reactive intermediate generation, analytical aspects and challenges, basic rudiments and advances in instrument capabilities of the ESI-MS technology is also covered and outlined. More information on dealing with the use of API-MS, in particular of ESI-MS to intercept transient species for the investigation of reaction mechanisms and dynamic processes in condensed phase can be obtained in some relevant reviews [2,21,24] and can refer to the recently published book “*Reactive Intermediates: MS Investigations in Solution*” edited by Santos. Hence the detailed reaction mechanisms of these successful examples are not considered, but a brief summary of the ESI-MS methods aiming at detecting reacting species within liquids will be emphasized. Furthermore, the state-of-the-art in ESI-based approaches and some new knowledge of the short-lived intermediates were reviewed quite lately in several excellent articles [4,25] that present the current thinking on direct linkages between gas-phase data and real chemical processes occurring in bulk solution. However, the purpose of the present review is to highlight the utility and expanding role of ESI-MS in probing reactive intermediates from various chemical reactions in solution, with special focus on the current efforts and progress in ESI-MS approaches using newly developed MS instruments and emerging ESI techniques. It is also worth emphasizing that the promise of combining other advanced characterization techniques and different strategies for improving method throughput, testing reality and real-time measurement will be discussed as well.

## 2. Reactive Intermediates Occurring in Liquid-Phase Reactions

According to the IUPAC definition, a reaction intermediate is a molecular entity (atom, ion, molecule, *etc*.) with a lifetime appreciably longer than a molecular vibration that arises (directly or indirectly) from the reactants and reacts further to give the final products of a chemical reaction. Reaction intermediates are often metastable species, and many intermediates are usually short-lived and highly reactive. However, more precise definitions like short-(fast) and long-lived (slow) are relative, e.g., some intermediate that are transient in one reaction mechanism can be considered stable or even live long enough to be detected, identified, isolated or used as reactants (or products) in other reactions. Reactive intermediates may be of various different chemical types, such as carbocations (including oxonium ions), carbanions (including enolates), carbenes, carbenoid, nitrenes (nitrenium ions), carbyne, arynes (benzyne, *etc*.), free radicals, radical ions (cations and anions), para-/ortho-quinine methides, tetrahedral intermediates, reactive oxygen species (ROS) and reactive nitrogen species (RNS).

In the broadest sense, transient intermediates can occur naturally within environments as diverse as living organisms and outer space, with the most striking properties such as short-lifetime, unstability (radioactivity), reactivity or even explosivity at ambient temperature. More specifically, reactive intermediate species during chemical processes mostly originate from (bio)chemical reactions and environmental degradations. In chemistry, except for a few concerted reactions in which all bond breaking and bond making occurs in a single elementary step, most chemical reactions occur in a stepwise manner with a complex sequence of steps via the formation and destruction of intermediates [12]. Since the non-concerted reactions, the dominant reaction type in chemistry, can usually proceed in multiple steps with an intermediate as the reaction product of each of these steps, the investigation of the existence of these reactive intermediates is a vast topic in (in)organic chemistry and can offer valuable information on the reaction pathway and mechanism. Thus, the knowledge of reactive intermediates in a common non-concerted process plays a key role in the understanding of reaction mechanism that represents the detailed, step-by-step description of a chemical reaction [2,3,27,28,29,30].

Another important pool of reactive intermediates is formed during metabolic transformation of xenobiotics (such as pharmaceuticals, pesticides and industrial pollutants) as well as endogenous compounds (such as steroids, prostaglandins and fatty acids) in biological systems [31,32]. Normally, the biotransformation reaction (oxidation and conjugation) catalyzed by various drug-metabolizing enzymes is generally considered as a detoxification process to facilitate elimination of the more active (toxic) and less hydrophilic chemicals from the body [33]. However, in some cases drug metabolism, especially oxidative biotransformation mediated by cytochrome P450 isozymes (CYPs) [34,35], can also convert chemically stable compounds into reactive metabolites (intermediates) followed by their covalent binding to biomolecules in cell, and therefore have the potential to initiate idiosyncratic adverse drug reactions or chemical-induced organ toxicities [4,36,37]. These reactive species generated through metabolic activation can be broadly classified into electrophilic intermediates (including both “hard” and “soft” electrophiles) and free radicals [36]. Although there is no definitive proof of a casual link between bioactivation of drug molecules and *in vivo* toxicity, circumstantial evidence currently suggests that the covalent modification of cellular macromolecules (such as DNA, RNA, proteins, carbohydrates and selective lipids) by chemically reactive intermediates may be recognized as a key mediator or initiator of drug-induced toxicity [5,38]. Simply, the basic principles of the chemistry involved in chemical-induced toxicity and possible detrimental effects may be described [31] as either (1) the irreversible reaction of an electrophile with a tissue nucleophile site in cellular constituents like proteins to form adducts, or (2) free-radical propagation especially prone to lipid peroxidation [39]. Since during normal metabolism, electrophilic metabolites and reactive oxygen species deriving from both endogenous and exogenous sources are capable of attacking biomolecules and inducing oxidative stress, DNA damage, cell proliferation or potential immune responses, structural characterization and quantification of reactive intermediary metabolites is critical to providing insight into the bioactivation mechanisms and for designing new drug candidates with improved toxicological profiles [4,33,36,40].

In addition, natural degradation and environmental transformation (including biodegradation [41] and photolytic destruction) as well as advanced oxidation process [26,42,43] of environmentally relevant contaminants (such as agricultural chemicals, industrial wastes, organic pollutants) may serve as a third main source of reactive intermediates. These degradation intermediates and reactive species produced are extremely powerful oxidizing species and may have possible increased risks to organisms or adverse ecotoxicological impact to human health [44,45].

## 3. Challenges for Analyzing Reaction Intermediates in Complex Systems

The understanding of the reaction pathway and mechanism is incomplete due to the experimental difficulties of monitoring reaction intermediates. To date, the capture/analysis of transient intermediates is extremely challenged by the following common features of reaction intermediates pertinent to (in)organic, organometallic chemistry and reactive metabolites of biological origin.

(1) Huge heterogeneity and variety in structural/chemical properties and molecular species for playing a broad and important role in inorganic and organometallic chemistry [30,46,47], organic and biochemical reactions as well as environmental degradations.

(2) Extraordinary high chemical reactivities of electronically unstable structures inclined to interact with other chemical species: in many organic reactions the most common types of reactive intermediates are often generated from chemical decomposition reactions, including both electron deficient species (not obeying the Lewis octet rule) such as carbocations and negatively charged carbanions; during bioactivation, many reactive metabolites are electrophilic and chemically unstable in aqueous solution under physiological conditions [4], undergoing further reaction with nucleophilic sites on biomolecules to form stable adducts.

(3) Typically short lifetime residence in the reaction mixture (not enough thermodynamic stability to be readily isolated and detected directly): reaction intermediates can vary widely in their chemical lifetime, from transient molecules with very short lifetime on the pico/nano scales (e.g., benzyne radicals [16], iminium ions in aqueous solution [48,49]), through semistable species on a lifetime scale of a few microseconds [10] or fractions of a second [8,50], to some long-lived intermediates within a duration of the order of seconds or minutes [51]; in a cell, the aqueous stabilities of reactive metabolites vary markedly between drugs [4], with apparent half-lives (*t*_1/2_) over a time frame normally ranging from fractions of a second to minutes or hours [31].

(4) Usually present only in extremely low concentration with respect to final stable reaction products: due to inherent reactivity/instability, most labile intermediates would not survive long enough for detection in the reaction mixture; owing to the electron-deficient nature for potentially covalent binding to any biological nucleophilic target present *in vitro/vivo* systems, most minor reactive metabolites of foreign compounds are not usually detectable in circulating blood [33,36,40].

(5) Broad diversity and dynamic changes for complex chemical reactions in condensed phases [2], particularly in various types of biological samples, which could only be achieved by rapid screening, real-time monitoring and more complete profiling of potential reactive intermediates [52] during the reaction process even at low levels in the solid state and/or in solution.

## 4. Current Strategies for Detecting Reaction Intermediates by ESI-MS

With the advent of soft API techniques, in particular ESI, API-MS has become unparalleled in its ability to characterize and determine a broad range of chemical or biological molecules. The ESI mechanisms and the fundamentals of ESI-MS have been discussed in some reviews as well as many original papers cited in this article. In the present review, only a brief description of the origins, philosophy, technology, and current application status of ESI-MS to investigate reaction intermediates will be given, and an attempt is made to define the technical requirements and different type of strategies for detecting intermediates and probing reaction mechanism.

### 4.1. Basics Principles of ESI-MS Detection of Reaction Intermediates in Solution Mixture

**Figure 1:**
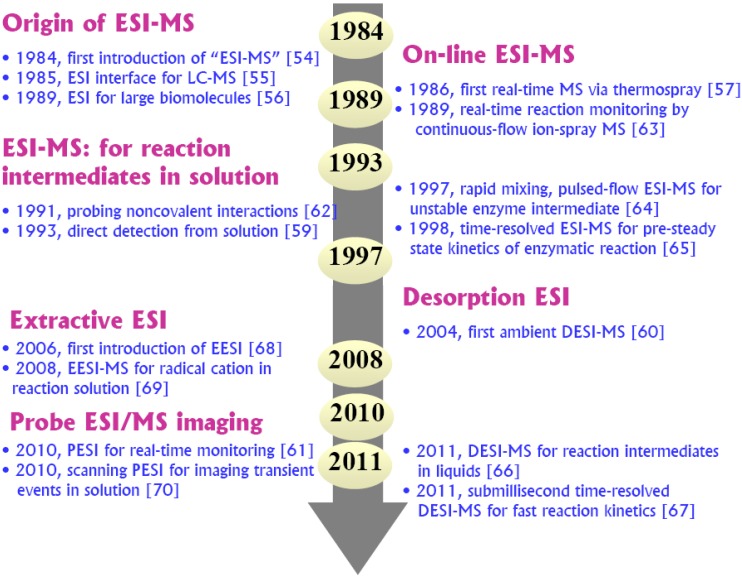
A historical timeline showcasing the significant events and milestones [54,55,56,57,58,59,60,61,62,63,64,65,66,67,68,69,70] in the development of ESI-MS for detection of reaction intermediates.

A decisive improvement in soft-ionization approaches to bridging the gap between the high vacuum and the “real world” of solutions was the emergence of a revolutionary API technology known as electrospray ionization (ESI). Following Dole’s initial studies on ESI [27,53] in the paint and polymer industry, Fenn and co-workers firstly introduced ESI as a leading technique for the mass spectrometric analysis of small ions, molecules [54,55] as well as biomacromolecules [56], for which the Nobel Prize was awarded in 2002. The first on-line API-MS investigation in chemical reactions was reported as early as 1986 [21,57,58]. In 1993, the validity (hypothesis) of using ESI-MS to detect and examine transient ionic intermediates directly from solution was successfully proved in organic reactions (Wittig, Mitsunobu, and Staudinger) to provide a mechanistic probe for the well-studied phosphine-mediated reactions [59]. More recently (since 2004 [60]), rapid developments in novel ambient ESI techniques, such as desorption electrospray (DESI), extractive electrospray (EESI) and probe electrospray (PESI), have demonstrated a promising potential for direct ambient sampling, *in situ* and time-resolved reaction monitoring on a reduced time scale [15,25,61]. After nearly 30 years of development, ESI-MS is widely accepted as the most useful and versatile means of creating access to the direct probing of reaction solutions, due to its unique power of gently transferring various (bio)chemical molecules from the condensed phase into the gas phase. Moreover, today, its capability is continuously evolving and adapting to the changing needs for monitoring fast/transient reaction process. As depicted in Figure 1, a brief historical timeline of ESI-MS development [54,55,56,57,58,59,60,61,62,63,64,65,66,67,68,69,70] for detecting reaction intermediates may offer some insight as to how big an impact of the ESI revolution on the well-established MS methods for studying reaction mechanisms in solution.

Given the above mentioned challenges, the experimental detection of short-lived and highly reactive intermediates is rather difficult and usually needs relatively sophisticated or fast reaction techniques. As to optical spectrometry methods, such as IR, UV and fluorescence, they can monitor intermediates *in-situ*, and sometimes can be very sensitive and selective. ESI-MS has been proved to be a valuable tool for proving the existence and establishing the molecular composition of transient species [30], owing to its outstanding unique superiorities [27] to fulfill the requirements.

Firstly, soft ionization can gently transfer intact molecular ions (especially key ionic intermediates) from a dilute solution directly to the gas phase, without inducing side reactions [26] to yield molecular fragmentation or cause biomacromolecular denaturation. It thus retains the charge states [3] and even weak nonconvalent interactions [62,71] in supramolecular complexes that actually exist in solution [53] during the transition into the gas phase. Because most chemical reactions occur in liquid phase while MS can only detect gas-phase ions, this feature (softness) of closely reflecting real-world condition and bringing MS down to the reaction environment, by “fishing” [72] solution ions directly into the gas phase (*i.e.*, generating intact gas-phase ions directly from electrolyte ions, large and complex species in solution) [19], is vital for the tremendous success and rapid broad use of ESI, particularly in the analysis of labile molecules or transient species originally present in the solution [27].

Secondly, ESI is the most commonly used API sources for a wide diversity of molecular species including polar molecules, supramolecules, biopolymers, inorganic and organometallic compounds. Although for some neutral molecules not easily ionized in solution, APCI seems to be a better alternative [21] for directly “fishing” neutral molecules (substrates and products) and even likely to preserve reaction intermediates and their coordination spheres [3,22,23] from solution to the gas phase, ESI is a softer process that normally releases ions preformed in liquid, in contrast to APCI occurring in the gas phase by strong heating of the nebulized eluent, and thus helps to preserve labile or transient species in the reacting solution [27,58]. In addition, some neutral species in solution can also be identified by means of covalent modification or chemical derivatization with charged substrate [72,73].

Thirdly, the elegant integration of efficient (rapid and direct) ESI ionization with powerful (sensitive and specific) MS detection for “ion-fishing” has become the dominant methodology to elucidate reaction mechanisms (kinetics) via ESI-MS(for ion identification and molecular profiling), and its tandem version ESI-MS/MS [13,24,27] (for structural characterization and simultaneous measurement) as well as ion-mobility (for shape-selective differention) [25] of reactants, products and key intermediates in the reaction mixture. Furthermore, ESI-MS experiments coupling with continuous-flow and rapid mixing devices have also proven to be amenable to on-line mechanistic investigations [3] and comprehensive snapshots [27] of the reaction progress, by real-time monitoring with millisecond time resolution of reactive intermediates even the short-lived ones occurring at very low concentrations [2,21,24]. In addition, the adaptation of a cutting-edge ionization technique (e.g., DESI) and the incorporation of a high-resolution (accurate) or other advanced MS(/MS) device (such as TOF, Orbitrap, FT-ICR) is a particularly attractive possibility for further broadening its interest and potential applications [61,70], due to the remarkably increased ability to identify ions directly sampled from a liquid-phase chemical reaction [14].

Lastly, ESI-MS can easily be combined with a variety of other complementary techniques. Recent combination of ESI-MS with advanced gaseous ion spectroscopies such as ultraviolet, vibrational spectroscopy and infrared-multiphoton dissociation(IRMPD) [29,74], powerful but time-consuming NMR experiments [14] not only brings additional prospects for establishing correlations between solution chemistry and the gas phase [25], but also offers the opportunity to directly probe the structure of putative reactive intermediates associated with real-world conditions, especially in the case of complex cascade reactions, many widely used analytical techniques such as NMR and IR spectroscopy often fail to isolate and characterize the reaction intermediates in solution [13].

### 4.2. Generic Tactics for Probing Reactive Intermediates in Solution

**Figure 2:**
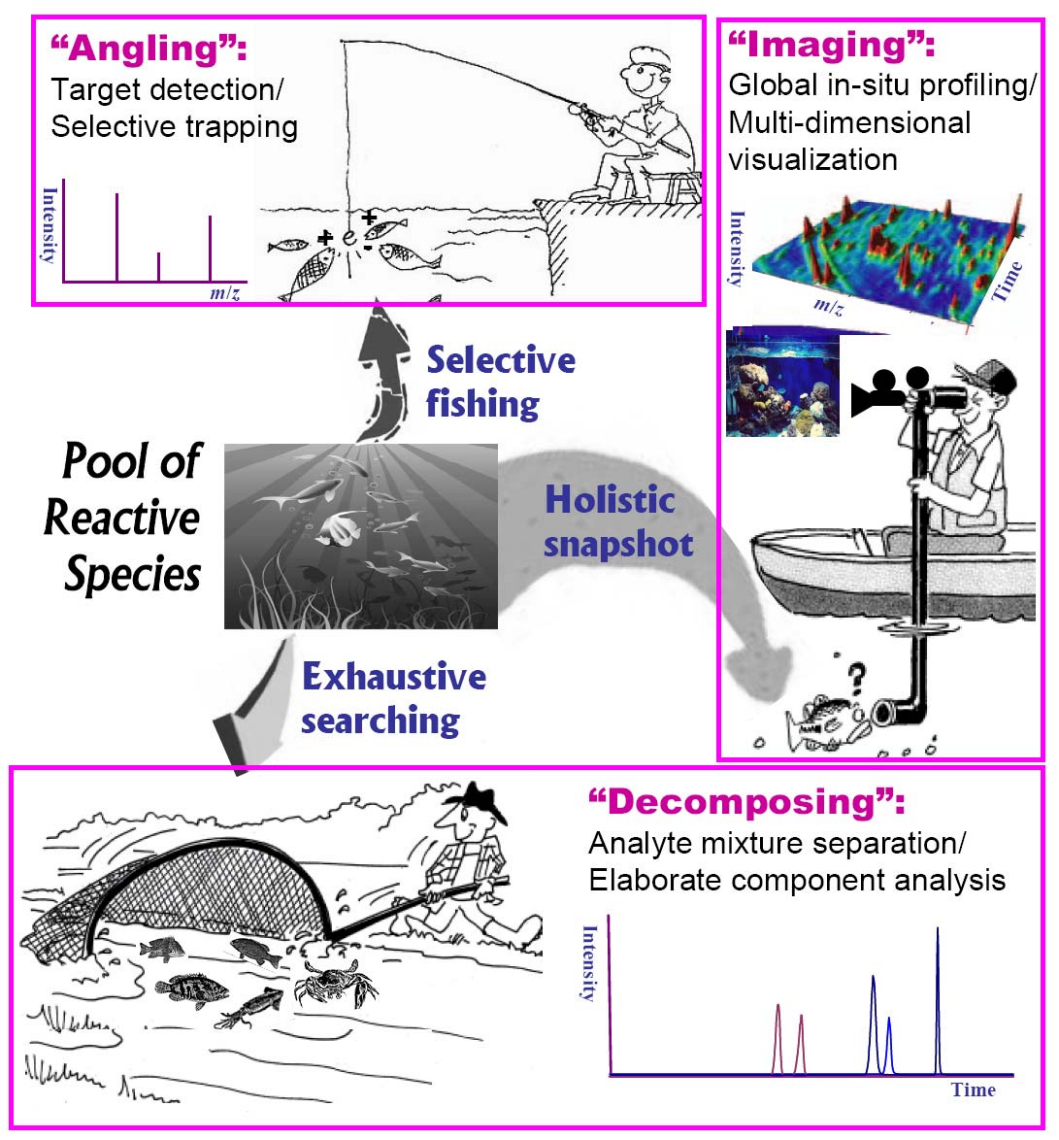
Sketch of generic ESI-MS strategies for probing reaction intermediates in solution: angling (selective fishing), decomposing (exhaustive searching) and imaging (holistic snapshot).

Generally, reaction intermediates often possess a dizzying array of structural and physicochemical properties that makes a single all-inclusive platform for probing total intermediates unlikely. Fortunately, many practical MS-based procedures and skills exist for dealing with analytical frameworks developed for intercepting transient intermediates in solution. In our latest review [75], we have categorized analytical methods for complex systems into three generic types: decomposition, angling and snapshot, based largely on their distinct performance characteristics including coverage, quality and speed. In this paper we continue with a detailed discussion of the proposed algorithm adapted for probing reaction intermediates in solution. Figure 2 depicts a pictorial view of these fundamental strategies based on ESI-MS for angling (selective fishing), decomposing (exhaustive searching) and imaging (holistic snapshot) of intermediate species in complex reaction medium.

ESI-MS angling, the so-called “ion-fishing” pioneered by Adlhart and Chen [72], is one of the most interesting methodologies for target detection or selective monitoring of ionic species in a complex mixture without any separation of individual components. However, ESI-MS was born with a genetic defect, *i.e.*, “blindness” to electrically neutral species [19]. A derivatization strategy that facilitates pulling out a desired species by way of selective labeling with charge substrates as probes (the “fishhook”) has proved as an encouraging solution to this limitation. By virtue of positive or negative charge tags, more neutral species such as radicals and carbenes [21,27,76] are fished directly from the reacting medium and fly “undisturbed” in the gas phase towards the MS detector [19]. Another successful application of the elegant strategy is chemical trapping by conjugation reactions for stabilisation of short-lived species to allow MS to manipulate more liable molecules, such as transient imine intermediates [2,77] and various types of reactive electrophilic metabolites in biological systems [36,37,78]. Thereby, the extraordinary ability to selectively “fish” ionic intermediate species (or neutral, zwitterionic or radical species in their ionic forms such as protonated, depronated, cationized or anionized molecules) from the solution allows a detailed overview of the reaction pathways, kinetics and mechanisms [24].

The “decomposing” strategy is based on component separation for the reaction mixture (Figure 2). In theory, any sample as a chemical mixture may be considered as the sum of a countable number of finite components that can be isolated and determined by efficient separation approaches [75]. Indeed, the use of high-resolution, multidimensional chromatographic separation and MS detection [33,79] is an indispensable element in hyphenated approaches towards the goal of “exhaustive” searching. It is evident that on-line and parallel combination of efficient separation-ionization-detection modes [80] can significantly improve the ESI-MS efficiency for expanding analyte coverage, *i.e.*, to detect as many reaction intermediates as possible.

ESI-MS imaging is emerging as a label-free diagnostic technique [81] that refers to rapid holistic characterization (detection, localization and visualization) of complex systems as an entirety based on unbiased methods, such as global intermediate/metabolite profiling and metabolomic approaches, without requiring target tracking or mixture fractionation procedures [75]. ESI-MS applications to chemical snapshot may be broadly divided into two categories (cartoon diagram in Figure 2): (1) simultaneous molecular profiling of comprehensive intermediate/metabolite species in a reaction solution, and (2) multidimensional integral mapping with rapid visualization of spatio-temporal distribution dynamics of a solution reaction. In the last decade, new progress in real-time ESI-MS techniques, such as direct sampling (or with little sample preparation), ambient ionization and non-destructive in-situ detection (or with minimizing destructive nature), has been exploring more possibilities to obtain the spatial distribution of reactive profiles directly from samples and to provide high-throughput, continuous snapshots of the dynamic ionic composition of complex reaction systems.

### 4.3. ESI-MS Monitoring of Intermediates in Reaction Process

Despite significant progress in the MS approaches for the study of chemical reaction mechanisms in the gas and condensed phases, the cornerstone in this research field for investigating intermediate species occurring in the reaction is their dynamic structural characterization. Compared with other spectroscopic methods, ESI-MS proves to be an excellent tool for simultaneous capture of intermediates and prompt determination of the composition (molecular mass and structural variation) of reaction mixture as a function of time, thus providing crucial insight into the transformation pathway, kinetics and the mechanism of (bio) chemical process [2]. It is now in most cases possible to inspect directly and characterize unambiguously of reactive intermediates [58] and monitor the real-time dynamic processes, based on the developments in the design and manufacture of direct sampling instruments, rapid mixing devices, stopped flow-quenched flow equipments and continuous on-line reaction apparatus, as well as their combinations to modern experimental schemes and ESI approaches. Many aspects of this topic have been the subject of several reviews to which the reader is referred [2,3,21]. In this section we will not make no attempt to supply any comprehensive review of their whole applications in reaction mechanistic studies, but will only present a brief and concise summary of ESI-MS approaches to probing of transient species in reaction mixtures as a function of time.

These approaches for monitoring of a reaction are arguably classified into two categories: (1) off-line consecutive sampling, and (2) continuous on-line reaction screening. The operation of off-line monitoring can be accomplished by taking aliquots from the reaction mixture at pre-determined time intervals during a reaction period, and then analyzing these aliquots immediately or after quenching and storing [2], to allow detailed structural identification of solution compositions even putative transient species (via intermediate complex [82] and reactive-metabolite trapping [77]), and accurate quantification for kinetics study by more reliable separation-based techniques (such as LC-ESI-MS).

To overcome the inherent limitations (discrete sampling, non real-time and inability to monitor transient species) of the off-line scenario, the on-line strategies [57,63], by connecting a reaction vessel of some type such as a syringe, capillary mixer, microreactor, photochemical chamber, electrochemical cell, *etc.* [21], directly to ESI-MS for a continuous transfer of reaction mixtures without intervention (including handling, quenching, and storage operations typical of off-line approaches) from the reactor to the ESI probe, allow the screening of the reaction and the direct monitoring of unstable intermediates in real time [2,3]. Moreover, a rapid-mixing/quenching device for direct interfacing with ESI-MS has provided easy access to the very early moments of a fast dynamic process in pre-steady-state kinetic investigations [64,65], which offers the key to the intimate details of a reaction mechanism [2]. Recent developments of microfluidic chip technologies and their direct coupling with ESI-MS detections [83,84,85] holds great appeal for further advancing the investigation of dynamic processes.

## 5. Recent Advances in ESI-MS Approaches to Probing Reactive Intermediates

Despite the great success of ESI-MS as a versatile technique to study reaction intermediates, the key bottleneck in current ESI-MS-based methodologies is the generally limited analyte coverage, throughput capacity and time resolution for direct on-line applications. Some new advances in ESI-MS-based methods for probing reactive intermediates/metabolites in solution are discussed below.

### 5.1. Wet-Chemical Treatments for Extending Detection Scope

Apart from instrument-dependent improvements in detecting reaction intermediates, the use of wet chemical techniques [75] to alter structural form of the analyte itself (via derivatization, hydrolysis, and isotopic exchange in solution-phase) or physicochemical properties of the sample (by solution additives), is also a simple alternative procedure for improving their performances in ESI-MS system. The common purposes of chemical derivatization [75,86] in ESI-MS are to achieve optimal chemical properties (such as polarity, ionizability, stability and fragmentability) for better sampling/separation, enhancing ionization efficiency, improving transient stability or/and increasing detection specificity.

As described above, the elegant combination of ESI plus “charge tag” (electronic label), the concept originating in the gas phase [25,87], has greatly expanded the applicability of ESI-MS for fishing neutral components in a reacting solution [72], and recently viewed as “charged wings for the flying fish of reaction intermediates” [19]. Charge-tagged substrates (by incorporation of a positive or negative charge-bearing group into the reactant for probing mechanisms [72]) or charged ligands (by replacement of a neutral ligand with a charged analogue for catalyst immobilization [88]) render more complex types of intermediates invisible to normal experiments amenable to manipulation using a standard ESI-MS technique. Another simple charge-tagging method for cationization of neutral species is the addition of traces of salts in the reacting solution sample, which leads to the corresponding alkali cation adducts of Ru-carbene [89], Lewis acid complex cations of radicals [12], or transition-metal complexes, for example, Ag-alkene π-complex [90]. Other chemical derivatization procedures [75] such as chemoselective labeling, isotopic tagging can also be used to transform neutral or non-polar molecules into ESI-MS-sensitive or ESI-MS-specific derivatives.

The idea behind the chemical trapping strategy is the use of wet-chemical reactions to stabilize transient intermediates that due to their high reactivity and short lifespan escape detection by traditional analytical methods. It has been widely applied to capture highly reactive metabolites and inherently unstable electrophilic intermediates originating from bioactivation of xenobiotics, followed by analyzing the stable trapped adducts that are readily amenable to traditional chromatographic separation and ESI-MS analysis. The often-used trapping reagent for reactive electrophilic species is glutathione (GSH), an abundant cellular thiol contributing significantly to effective detoxification due to its highly nucleophilic properties of the thiol moiety. Other nucleophiles including thiol-containing compounds (GSH derivatives and *N*-acetylcysteine), amines (semicarbazide and methoxyamine) and cyanide are also usually used as nucleophilic trapping agents [36]. Recently, more synthetic GSH-derivatives with a large structural variety, such as isotope-labeled glutathione (GSX) [91], fluorescence-tagged GSH (Dansyl-GSH) [92], quaternary ammonium GSH analogue (QA-GSH) [93], bis-methyl glutathione ester (GSH(CH_3_)_2_) [80], bi-functional γ-glutamylcysteinyllysine (γGSK) [94], *N*-(2-bromocarbobenzyloxy)-GSH (GSH-Br) [95], and ferrocenylpropionate glutathione (FP-GSH) [96], have been developed for improvements in detection selectivity, ionization efficiency, chromatographic resolution and reactivity towards different types of reactive species, for fragment-specific structural identification, as well as for quantitative assessment of reactive metabolite formation [40]. In addition, some neutral components or unstable metabolites in a reacting mixture can be sampled efficiently and additional insights into the transformation pathways can be obtained by using solution additives (such as electrolyte, reaction promotor, protectant and stabilizer) to change the ionization conditions [97] or pre-ionization process [98,99].

Table 1 gives a summary of some selected wet-chemical techniques that have been combined with ESI-MS to detect reactive intermediates originating from chemical and biological reactions. The list is not at all comprehensive, but presents a brief overview about the scope of wet chemistry for ESI-MS probing reacting species in solution. Some representative examples of this topic have been discussed extensively elsewhere [27,33,88] are also included in Table 1.

**Table 1:** Selected examples of wet-chemical techniques for ESI-MS detection of reactive species.

Reactive species	Origin	Wet chemistry reagent	Intended use	Reference
Monophosphine norbornene complex	Ring-opening metathesis polymerization (ROMP) of norbornene	Norbornene-CH_2_P^+^Ph_3_Cl^−^; -CH_2_N^+^(CH_3_)_2_(CH_2_Ph) Cl^−^ (as charge-tagged substrate)	Charge tag (+)	[53,72]
Pd-containing intermediates	Cu-free Sonogashira (Heck alkynylation)	[*p*-IC_6_H_4_CH_2_PPh_3_]^+^[PF6]^−^ (as charge-tagged substrate)	Charge tag (+)	[100]
Organopalladium species	Pd-mediated (Heck) cross-coupling reactions	[*p*-IC_6_H_4_N(CH_3_)_3_]^+^[I]^−^ (as charge-tagged substrate)	Charge tag (+)	[101]
Bis(phosphino) palladium species	Pd-catalysed (Sonogashira) cross-coupling reaction	[Na]^+^[PPh_2_(*m*-C_6_H_4_SO_3_)]^−^ (as charge-tagged substrate)	Charge tag (−)	[102]
Palladium complex	Suzuki and Heck Phosphine-Free Reactions	Acetate anion bearing an imidazolium cation (ligands)	Charge tag (+)	[103]
Neutral radicals	Radical chain reactions	Lewis acids: Sc(Otf)_3_	Cationization as [R∙Sc(Otf)_2_]^+^	[12,104]
Ru-carbene species	Ru-carbene based olefin metathesis	Alkali-metal salts	Cationization as alkali adducts	[89]
Pd hydride; Neutral Pd(II) complex	Pd-catalyzed addition of allenes to organoboronic acids	CH_3_COOH	Facilitating ESI (–e and −H∙) to form cationic Pd complex	[97]
Hydroxyl-sulfonamide	Metabolic bioactivation of sulfonamide	Ascorbic acid	Stabilizer to inhibit oxidation	[99]
Reactive metabolites	Electrochemical simulation of oxidative metabolism	Ferrocenylpropionate (FP)-GSH	Trapping agent; Retention tag	[96]
Reactive drug metabolites	P450-mediated drug bioactivation	Deuterium labeled bis-methyl GSH esters (GSH(CD_3_)_2_)	Trapping agent; Ion-signal sensitizer	[80]
Bioactivated intermediates	Bioactivation of xenobiotics	D-Isomer of peptide: gly-tyr-pro-cys-pro-his-pro	Trapping agent	[105]
Radical intermediaries	(Glyco-)xidation of phosphatidylethanolamine	5,5-dimethyl-pyrroline *N*-oxide (DMPO)	Spin trap	[106]
Electrophilic species	P450-mediated drug bioactivation	*N*-(2-bromocarbobenzyloxy)-GSH (GSH-Br)	Trapping agent	[95]
Epoxide metabolites	*in vitro* metabolic bioactivation	Cob(I)alamin	Trapping agent; Charge tag	[107]
Reactive metabolites	P450-mediated drug bioactivation	Stable isotope labeled GSH, KCN and semicarbazide	Trapping agents; Isotopic tagging	[108,109]
Reactive metabolites	UGT-mediated drug bioactivation	*N*-acetylcysteine (NAC)	Trapping agent	[110]
Reactive metabolites	P450-mediated drug bioactivation	quaternary ammonium GSH conjugating agent (QA-GSH)	Trapping agent; Semiquantitation tag	[93]

### 5.2. Current Evolutions of Time-resolved ESI-MS for Complex Solutions

Notwithstanding the success of ESI-MS for direct ion-fishing and, via numerous clever wet-chemistry strategies, for on-line or off-line monitoring of transient intermediates/metabolites from fast reactions in solution, instrumental improvements in real-time reaction sampling device, on-line interface design or/and fast detection with high temporal resolution are always more important to achieve them. Up until now, many different experimental methods have existed for on-line kinetic studies of (bio)chemical liquid-phase processes, and by coupling a continuous-flow mixing apparatus directly to an ESI source, several time-resolved ESI-MS methods have been established for obtaining MS data in kinetic and in spectral mode with the time resolution from seconds to milliseconds [63,64,65,66,67,111]. The first successful application of time-resolved ESI-MS for measuring the pre-steady state kinetics of an enzymatic hydrolysis on a time scale of tens of milliseconds involved direct observation of a transient enzyme intermediate [65].

Another promising approach to overpass the “communication barrier” between the real word of solution reactions and MS detection performing in high vacuum [19,112] is the direct sampling ionization of reacting solutions in their native states by means of emerging ambient ionization techniques. The advent of the first ambient ionization technique, known as desorption electrospray ionization (DESI), was pioneered by Cooks’ research group in 2004 [60]. This new DESI method that permits direct ionization of a trace sample in its native environment without need for sample preparation is a combination of atmospheric pressure electrospray ionization (ESI) with a desorption ionization (DI) technique. Traditionally, DI methods such as MALDI and secondary ion mass spectrometry (SIMS) are critical for producing intact molecular ions from condensed-phase samples (surfaces) under vacuum conditions. Later, the atmospheric pressure version of MALDI (AP-MALDI) became an important progenitor of the ambient MS technology, and accordingly DESI can be considered an atmospheric pressure version of SIMS [113,114] to integrate the benefits of ESI, SIMS and DI [60]. Since the introduction of the direct ambient DESI, more than 30 ambient ionization approaches have emerged [113], including ESI-modified (spray-based) and APCI-modified (electric discharge-based) setups [115,116]. As a representative ambient ionization method, DESI is one of the most prominent and widely used tools for direct, rapid and non-destructive analysis of untreated samples in open air under ambient conditions. It is carried out by directing a stream of fast-moving electrosprayed charged solvent droplet towards the sample surface of interest, from which it picks up the analytes and propel the resulting secondary microdroplets (containing desolvated ions formed or originally present on the surface) through the atmosphere into the mass analyzer [113,114]. The novel feature of DESI make it possible for unimpeded access to a variety of sample types with minimal or no pretreatment. In the last few years, a multitude of different spray-based versions of DESI (Table 2) have been reported like electrospray laser desorption ionization (ELDI), fused droplet electrospray ionization (FD-ESI), matrix-assisted laser desorption electrospray ionization (MALDESI), reactive DESI, extractive electrospray ionization (EESI), neutral desorption extractive electrospray ionization (ND-EESI), laser ablation electrospray ionization (LAESI), infra red laser ablation electrospray ionization (IR-LAESI), laser-assisted desorption electrospray ionization (LADESI), laser desorption electrospray ionization (LDESI), laser-induced acoustic desorption-electrospray ionization (LIAD-ESI), desorption electrospray metastable-induced ionization (DEMI), probe electrospray ionization (PESI), and liquid micro-junction surface sampling probe/electrospray ionization (LMJ-SSP/ESI) [115,116].

**Table 2:** Glossary of different ESI-related methods listed in order of publication. Abbreviations for full name of ionization techniques and references to the original work are provided within the text.

ESI type (Acronym)	Date of origin	Ionization principle	Significant feature	Reference
ESI	1984	Electrospray ionization	API for ions in solution	[54]
DEP	1999	Direct electrospray probe	Electrospray without capillary	[117]
FD-ESI	2002	Fused-droplet electrospray ionization	Extremely high salt tolerance	[118]
DESI	2004	Desorption electrospray ionization	Direct ambient MS sampling	[60]
ELDI	2005	Electrospray laser desorption ionization	Additional selectivity and scope	[115]
EESI	2006	Extractive electrospray ionization	Liquid extraction between two sprayers	[68]
Reactive DESI	2006	Reactive desorption electrospray ionization	On-line reaction for specific identification	[114]
PESI	2007	Probe electrospray ionization	Solid needle for non-invasive ESI	[61,119]
MALDESI	2007	Matrix-assisted laser desorption electrospray ionization	Shot-to-shot reproducibility; Limitations in spatial resolution	[115]
LAESI	2007	Laser ablation electrospray ionization	3-D imaging biomolecular distributions	[120]
ND-EESI	2007	Neutral desorption extractive electrospray ionization	Direct ionization of nonvolatile analytes inside a heterogeneous or viscous matrix	[121]
IR-LADESI	2008	Infrared laser-assisted desorption electrospray ionization	Direct analysis of water-containing samples under ambient conditions	[115]
DEMI	2009	Desorption electrospray metastable-induced ionization	Direct multimode detection of intact molecules	[116]

### 5.2.1. Ambient ESI for Direct Ionization of Liquid Samples

In DESI, a nebulizing gas/charged microdroplets of solvent (like methanol, acetonitrile, water) generated by traditional ESI process is used to create a high-velocity spray for desorbing condensed-phase and surface-bound analytes as secondary ions. DESI is a versatile interface for direct extraction/ionization of the surface analytes from the dried samples, and allows for two-dimensional (2-D) molecular imaging of the solid surfaces. But for liquid samples, they are typically required to be dropped, adsorbed or dried on a substrate (cotton swab, filter paper or membrane, *etc*.) in air before the DESI experiments, wherein the liquid samples could be blown away from the surface [122,123]. Recently, some new experimental attempts and improved procedures has been reported for direct analysis of liquid samples by DESI [113,123], which holds great promise for DESI to monitor transient intermediates during the chemical reactions. To overcome the issue of liquid splashing, Zhang and coworkers constructed a multichannel device (with 16 parallel capillaries) coupled with DESI-MS, by which the liquid sample in capillary was driven out with the nebulizing gas, sampled, ionized, and then analyzed in a high-throughput fashion [124]. Another work on liquid-phase DESI by Chen and Miao used a syringe pump to continuously drive the solution through a silica capillary to needle tip for direct desorption/ionization [123].

EESI, introduced by Chen and colleagues in 2006 [68], a variant on the basic FD-ESI experiment [118], is another popular spray-based ambient ionization method suited for online liquid sampling and direct ionization of complex solutions, biomatrices, aerosols and suspensions in real time without any sample preparation [125]. In EESI, the (neutral) analytes in a raw sample are continuously infused through a sample introduction channel, and then ionized by an online collision/extraction with the primary charged droplets generated from the traditional ESI spray channel. In contrast to the 2-D surface desorption ionization in DESI, EESI with 3-D space dispersed extractive ionization, does not suffer from ion suppression by dispersing the matrices over a large 3-D volume, and also does not require any sample preparation, to minimize the speciation changes and chemical contaminations and physical damages caused by a harsh environment, because the nebulization and ionization processes are safely isolated in both space and time [68,125]. These unique features makes EESI an ideal ionization method for rapid, on-line monitoring of (bio)chemical reactions from complex liquid samples [69,126,127].

A furthermore modification of the EESI-MS method by using a neutral desorption (ND) sampling gas beam [121], termed ND-EESI-MS, allows one to gently and efficiently acquire analytes from complex biological surfaces [128] and to easily detect and measure volatile and nonvolatile analytes from highly viscous samples [129]. The ND sampling process is totally separated from the extractive ionization processes that occurs in a 3-D space without contacting with the bulk sample, and enables continuously liberate analytes from virtually any type of surface without substantial sensitivity loss for subsequent EESI-MS analysis. Actually, EESI-based method is not merely a tool for surface analysis because (1) for homogeneous solutions, the sampling fraction represents the molecular composition of the whole solution sample [130]; (2) with the ND sampling device, liquid droplets reflecting the chemical constituent of the bulk solution sample can also be liberated from inside a complex heterogeneous liquid or viscous matrix for direct EESI ionization [129]. These advantages make ND-EESI-MS to broaden the EESI application, tolerate extremely complex matrices, and realize real-time chemical profiling of the complex samples under ambient conditions.

### 5.2.2. Desorption ESI-MS for Monitoring Fast Reactions and Intermediates

In traditional ESI-MS-based “mixing-sampling-ionization-detection” for reaction monitoring and solution characterization, the possibility to increase its total throughput depends to a great extent on three factors: on-line/real-time sampling, direct/ambient ionization and rapid/high-resolution detection. As remarked above (in Section 4.3), many rapid-mixing/sampling devices and continuous-flow direct infusion methods are a simple and efficient means for on-line monitoring of dynamic process of chemical reactions in solution. More powerful MS analyzers (high-resolution [131], multi-stage tandem version [132], fast scanning and polarity switching) with advanced data-mining strategies [33,36,37,40,79] make it possible to large-scale screening, identification, and quantification of (un)target reactive analytes with high degree of sensitivity, accuracy and precision, and thus particularly useful for probing complex cascade reaction processes [13,133].

With the emergence of new ambient DI techniques, promising developments in DESI-based methods allow for monitoring of liquid samples on a substantially reduced time scale. It can also be combined with a relatively simple continuous-flow setup to extend their applications for on-line monitoring of the changes of intermediate species from chemical reactions [15,123,126,127,134,135]. Another development of DESI, called reactive DESI [136], with the aid of chemical derivatization by using a specific reagent added into the spray solution during the sampling process (particular ionic reactions accompanying desorption), has proven an interesting approach to tracking short-lived intermediates (as characteristic adduct ions for confirmatory identification) without sample pretreatment [50,66,137]. In reactive DESI experiments, the addition of different types of reagents in the spray solution (reactive solvent) are intended to allow selective interfacial ion/molecule reactions with the target analyte(s) on the sample exterior in the open atmosphere to yield gas-phase ions [114] of the derivatized products. This type of reactive desorption/ionization has gained increasing attentions since it represents an easy way to add chemical selectivity and expand the scope of the ion chemistry performed in the basic DESI experiments. There are a wide range of selective reactions types, such as simple ion adductions, redox reactions, noncovalent complex formations, charge labeling and functional group modifications available for improving ionization efficiency and enhancing detection sensitivity and selectivity [113,114,123,138]. Similar to reactive DESI, a reactive form of EESI has also been explored to improve the sensitivity and specificity of EESI for efficient detection of diethylene glycol in viscous toothpaste [139].

In terms of time-resolution, in most scenarios, the conventional ESI-MS techniques can only probe reactive species with relative long lifetimes, while DESI-based methods with direct sampling ionization remarkably increase the temporal resolution for detecting reaction intermediates in liquids. Taking advantages of the fast ambient ionization and high-resolution MS detection, current ambient ESI-MS can further advance their skills and talents in sniffing out transient reactive species [14] on rapid time scales typically down to milliseconds. To date, a few examples of successful applications include detection of distonic tetramethylene radical cation intermediates from fast electron-transfer-catalyzed dimerization by EESI-MS/MS at ambient conditions [69], and interception of transient intermediates in the early phases of Ru(II)-promoted hydrogen transfer catalysis [50,66] and Eschweiler-Clarke reaction [137], by reactive DESI experiments coupled with ion trap tandem MS or high-resolution Orbitrap analyzer.

Since the reaction starts when the droplets of spray (or contained reagent) hit analytes on the surface, it is interesting to directly “observe” these unstable intermediates formed in the first few milliseconds of the chemical reactions under the ambient conditions. The principle underlying these methods for acquiring unique intermediates and such high time-resolution spectral data not seen with the conventional ESI-MS in chemical reactions can be explained by some recent experimental measurements and theoretical investigations. (1) A short distance between the surface impact site and the MS inlet (typical 5 mm) [140] and a relatively high velocity of the secondary microdroplets leaving from the surface to the inlet (*ca.* 4 m/s) [140,141] results in the reaction proceeding only for a few milliseconds [66] (limited to <2 ms timescale) during the desorption/ionization, evaporation and transfer through the surface-to-inlet distance, before the chemical reaction is quenched by the formation of gas-phase ions [14]. (2) Another exciting feature is the remarkable acceleration of a certain chemical reaction within the evaporating DESI microdroplets as compared to that obtained in bulk solution, probably due to a gradient increase in physicochemical properties [137,142]. In DESI, the chemical reactions would occur more quickly in the liquid-phase environment of the secondary microdroplets (2–4 μm in diameter [140]), which allows the reaction intermediates produced in microscale droplet volumes (approximately 4–33 mm^3^ [66]) to be probed in real time by DESI-MS on a very short timescale (on the order of milliseconds). In particular, the short time scales in microdroplets from DESI enable detection of certain reactive species with high sensitivity (pmol quantities), and even interception of some new transient intermediates that cannot be observed with traditional ESI-MS analyses conducted in bulk reaction vessels because the reaction time is much longer (e.g., tens of seconds) than that in the desorbed microdroplets of DESI [66]. (3) Further time-resolution improvement can be achieved with a newly reported time-resolved liquid jet DESI-MS with submillisecond time-resolution (300 μs) for very fast (bio)chemical kinetic study [67], based on a direct, fast and time-dependent ionization of the analytes in a high-speed “flying” liquid jet stream, by increasing the liquid jet speed (e.g., 100 m/s) or decreasing the jet sampling distance (e.g., 0.1 mm). The free high-speed jet stream is generated through a rapid mixing of two separate reactant solutions and then ionized and monitored in real time by DESI-MS at different jet distance intervals corresponding to the different reaction times.

Table 3 shows a series of selected applications, not exclusively but mostly with time-resolved ESI-MS for monitoring fast reactions and intermediate species, in the hope of exemplifying the diversity of apparatus and methods that can be used for probing the mechanisms of various (bio)chemical reactions. Also the readers are directed to a representative compendium of the references cited (in Table 3) for more details about the information. There is increasing evidence demonstrating the great capacity of desorption ESI-MS using DESI microdroplets as reaction vessels for monitoring reactive intermediates directly in solution undergoing fast reactions and consequently suited for interpreting reaction stoichiometry, kinetics and mechanisms in a time window of milliseconds. But despite the obvious attraction of such real world MS investigations to open a new route for probing novel reaction intermediates and mechanistic scenarios via short-lived liquid-phase transients of reactions in desolvating droplets, a general question remains unexplored about the suitability of using the ESI droplets as a microvessel for direct investigation and prediction of practical (bio)chemical reactions occurring in bulk solution [19,69]. It is important to note that chemical reactions can be drastically accelerated [142] in highly charged and concentrated droplets, even unexpected oxidation [143,144] or new surprising and promising reactions [19] can be revealed and monitored by microdroplet fusion during DESI. Hence it seems likely that an unknown perturbation of the situation in solution associated with ESI process [25] will continue to be the crucial challenge to bring these gas-phase reactions down into the solution-phase properties [19,113]. Nevertheless, as far as chemical reaction is concerned, there exists a close correlation between ESI-MS data and condensed-phase chemistry in terms of reactant concentrations, reaction times and physicochemical conditions [25]. Recent developments in MS technology are offering additional prospects for establishing more direct linkages [25] between ESI-MS measurements and solution chemistry “at the molecular level with unsurpassed speed, selectivity, sensitivity, ease and flexibility” [19].

**Table 3:** Representative applications of time-resolved ESI-MS for monitoring fast reactions and intermediate species.

(Bio)chemical reaction	Intermediate species	Instrumentation (reaction-sampling-ionization-detection)	Temporal resolution	Reference
Synthase catalyzed reaction	Tetrahedral intermediate	Pulsed flow (rapid-mixing) device—ESI-QMS	30 ms	[64]
Pre-steady state enzymatic kinetic	Transient enzyme intermediate	Two syringes in a reaction mixing tee—ESI-QMS	tens of ms	[65]
Chlorophyll demetalation	Specific reactive species (time profile)	Capillary mixer with adjustable reaction chamber volume—ESI-QQQ	ms	[111]
Pd(PPh_3_)_4 _decomposition	Pd-containing reactive species	Continuous pressurized sample infusion—ESI-QTOF	N/A	[145]
Sandmeyer’s cyclization	Three new cationic intermediate	Microreactor—ESI-QTOF	subs	[146]
Electro-oxidation	Perylene radical cation	Electrochemical cell—DESI-QTrap	N/A	[123]
Pyrolytic reactions	Reactive ketenes	Flow pyrolyzer-multichannel ESI-QQQ	~0.2 s	[147]
Electron-transfer cata-lyzed dimerization	Distonic tetramethylene radical cation	Gas/liquid setup—EESI-QTOF; Liquid/liquid setup—EESI-QTOF	ms	[69]
Catalytic transfer hydrogenation	Ru-complex intermediates	Microdroplet reaction vessel for reactive DESI-IT	ms	[66]
Catalytic transfer hydrogenation	Transient Ru-methyl formate species	Microdroplet reaction vessel for reactive DESI-Orbitrap	subms-ms	[50]
Morita-Baylis-Hillman reaction	Two key MBH intermediates	Venturi easy ambient sonic-spray ionization (V-EASI)-QMS	N/A	[135]
KDO8P synthase reaction	Noncovalent acyclic hemiketal intermediate	Rapid mixing device—ESI-QTOF	50−630 ms	[148]
Eschweiler-Clarke reaction	Reactive iminium ion; Sodiated amino alcohol	Reactive DESI-IT	ms	[137]
Fast oxidations of I^−^ and S_2_O_3_^2−^ by O_3_	Short-lived ISO_3_^−^ and IS_2_O_3_^−^	Reactive DESI-QMS	~1 ms	[149]
Catalytic acetylation of benzyl alcohol	Positively charged intermediates	Online ND setup—EESI-QTOF	<1 s	[126]
Borsche-Drechsel cyclization	Ionic intermediate (protonated hydrazone)	Electrosonic spray ionization (ESSI)-IT	N/A	[150]
Zemplén deprotection	Mono-deprotection intermediate	Capillary action supported sampling tool—contactless API emitter-IT	1 min	[15]
Electrochemical red/oxidization	*N*-hydroxyl and amine labile intermediates	Electrochemical flow cell—nanoDESI-LTQ/Orbitrap	N/A	[151]
Schiff base formation	Hemiacetals	PESI-QTOF	0.1−0.33 s	[61]
Monitoring of 3-D cell culture system	(Bio)chemical transients	Inline microdialysis—ambient nanoESI-QTOF	100 μm (spatial)	[152]

### 5.3. Emerging ESI-MS-Based Reaction Profiling and *in-Situ* Imaging

Based on ESI-MS screening of GSH-trapped reactive metabolites [33,36,40], a novel metabolomic approach has been currently developed as an efficient tool for large-scale fishing reactive metabolites from a complex biological matrix, and untargeted profiling xenobiotic bioactivation, especially for interpreting adducts generated via uncommon metabolic pathways [153].

With the emergence of novel ESI-MS approaches such as direct sampling, ambient ionization and rapid detection with high spatiotemporal resolution, non-destructive *in-situ* MS characterization, an exciting field of molecular imaging, is exploring more and more possibilities for high-throughput direct analysis of bio-samples and visualizing spatio-temporal dynamics of comprehensive profiles of reaction intermediates. The open-air EESI technique and typical ND-EESI setups have been applied for the rapid *in vivo* fingerprinting of complex surfaces and living objects at molecular levels in their native states [128,129,154].

Whereas classical MALDI and SIMS have been primarily used for profiling/imaging of tissues and cells, there is growing interest in applying ambient surface ionization MS to imaging analysis [81,113,116]. Spatially localized sampling is one of the key elements of imaging techniques for their spatial resolution. At present, the state of the art for ambient imaging MS of biological tissues is typically on the order of 100–500 μm [155], and most DESI imaging is performed with a spatial resolution of 180–220 μm [113]. Recently, ambient ESI-based, untargeted chemical imaging of dynamic events with enhanced resolution has been achieved by using a sampling probe [70] or nanoESI device [152,155] that allows continuous sampling from a highly localized volume of a complex solution. Basically, since the sampling area is determined by the needle probe dimension, reducing the sampling capillary radius can improve both spatial and temporal resolutions [70]. PESI is based on a technique called direct electrospray probe (DEP) reported by Shiea *et al.* in 1999 [117]. In PESI [61,70], a solid fine needle (e.g., 20 μm) is used as the sampling probe and the ESI emitter to obtain transient images of (bio)chemical events originating from a live sample. Furthermore, some latest versions of nano-DESI [152,155,156,157], in conjunction with high-resolution MS detections, can also open up a new ESI-MS ambient imaging tool for sensitive, high-throughput, comprehensive, quantitative and *in-situ* characterization and high spatial-resolution profiling of biological samples without sample pretreatment.

## 6. Conclusions

Reaction is the “soul” of chemistry. In reaction schemes, “unobserved” intermediates were first hypothesized more than a century ago and only indirect evidence points to their existence because of their high reactivity [158]. The characterization of reactive intermediates is central to mechanistic and quantitative understanding of modern chemistry and a molecular view of biology. Conventional methods for probing reaction mechanisms in solution include physicochemical measurement, chemical labeling/trapping and continuous dynamic monitoring with classic spectroscopic techniques. From its introduction by Fenn *et al*. in the mid-1980s, ESI-MS technology has taken an irreplaceable position with a myriad of previously unthinkable routes to probing reaction mixtures and mechanistic details in chemistry, biology and related sciences.

New versions of ESI-MS using a fascinating ambient interface, are emerging as promising means under active investigation of fishing and probing reacting intermediates and other transient species directly from a liquid reaction mixture. However, the limitations of using these DESI-related methods in detecting reaction intermediates should be pointed out. For examples, the ionization methods may not be good analytical methods for some organic reactions when: (1) the solution contains metal catalytic particles; (2) the solution is made by pure less polar or non-polar solvent; (3) the reaction must be performed at elevated temperature; (4) the reaction must be performed in a reflex system. Moreover, it is difficult to differentiate gas-phase reaction products generated in the ion source from the intermediates in reactive solution probably because they could be easily fragmented or recombined during the ionization and flight to MS detector. So the possibility of additional pathways shouldn’t be overlooked and the corresponding methods and strategies should be developed to solve the problem.

Current methodologies and strategies for obtaining real-time, non-destructive *in-situ* information about reaction intermediates in solution have greatly benefited from the increasingly progress in miniaturization and seamless integration of continuous-flow system (such as rapid-mixing, direct sampling and microfluidic chip-based automation), ambient ESI designs as well as high-resolution, rapid response MS analyzers. In the future, as (bio)chemical investigations become ever more complex (e.g., multidimensional high-throughput reaction discovery [159], large-scale molecular networks within a cell [14]), exciting advances of ESI-MS-based techniques can be anticipated to further extend their roles in time-resolved spatial profiling of reaction intermediates, *in-situ* localization quantification of reactive metabolites in intact tissues, and high-resolution chemical imaging and multi-dimensional spatial arrangement [157] of short-lived species and their dynamic evolution in the condensed phase.
